# Unveiling the Protective Role of Melatonin in Osteosarcoma: Current Knowledge and Limitations

**DOI:** 10.3390/biom14020145

**Published:** 2024-01-24

**Authors:** Nojoud Al-Ansari, Samson Mathews Samuel, Dietrich Büsselberg

**Affiliations:** 1 Department of Medical Education, Weill Cornell Medicine-Qatar, Education City, Qatar Foundation, Doha P.O. Box 24144, Qatar; naa2070@qatar-med.cornell.edu; 2 Department of Physiology and Biophysics, Weill Cornell Medicine-Qatar, Education City, Qatar Foundation, Doha P.O. Box 24144, Qatar

**Keywords:** anti-cancer therapeutics, apoptosis, bone cancer, melatonin, osteosarcoma

## Abstract

Melatonin, an endogenous neurohormone produced by the pineal gland, has received increased interest due to its potential anti-cancer properties. Apart from its well-known role in the sleep–wake cycle, extensive scientific evidence has shown its role in various physiological and pathological processes, such as inflammation. Additionally, melatonin has demonstrated promising potential as an anti-cancer agent as its function includes inhibition of tumorigenesis, induction of apoptosis, and regulation of anti-tumor immune response. Although a precise pathophysiological mechanism is yet to be established, several pathways related to the regulation of cell cycle progression, DNA repair mechanisms, and antioxidant activity have been implicated in the anti-neoplastic potential of melatonin. In the current manuscript, we focus on the potential anti-cancer properties of melatonin and its use in treating and managing pediatric osteosarcoma. This aggressive bone tumor primarily affects children and adolescents and is treated mainly by surgical and radio-oncological interventions, which has improved survival rates among affected individuals. Significant disadvantages to these interventions include disease recurrence, therapy-related toxicity, and severe/debilitating side effects that the patients have to endure, significantly affecting their quality of life. Melatonin has therapeutic effects when used for treating osteosarcoma, attributed to its ability to halt cancer cell proliferation and trigger apoptotic cell death, thereby enhancing chemotherapeutic efficacy. Furthermore, the antioxidative function of melatonin alleviates harmful side effects of chemotherapy-induced oxidative damage, aiding in decreasing therapeutic toxicities. The review concisely explains the many mechanisms by which melatonin targets osteosarcoma, as evidenced by significant results from several in vitro and animal models. Nevertheless, if further explored, human trials remain a challenge that could shed light and support its utility as an adjunctive therapeutic modality for treating osteosarcoma.

## 1. Introduction

Melatonin, an endogenous product of the pineal gland, has emerged as an interesting biomolecule implicated in several biological/physiological processes. As a naturally occurring hormone, it is well studied and known for its role in regulating the circadian rhythm and the sleep cycle in humans. Scientific evidence points to the role of melatonin in lipid metabolism, thermoregulation, energy metabolism, and immunity and its vitality in maintaining cardiovascular, reproductive, and neurological health [1,2]. Studies have also explored its role in bone metabolism, showing that it could regulate mesenchymal stem cell (MSC) differentiation and support osteoblastic growth and mineralization [3,4]. Curiosity regarding other effects of melatonin that remained unknown has led many scientific/research initiatives to explore the multifaceted potential of melatonin.

The latest research has shifted towards investigating the oncostatic characteristics of melatonin showcased in different malignancies, including breast, ovarian, lung, and prostate cancers [3,4]. Such budding potential, along with the variable nature of melatonin production and physiological levels that decline in the pediatric population near puberty, coincides with a rise in osteosarcoma (OS) incidence in this population [3,5]. This promoted inquiries on the osteosarcoma-centered outcomes of melatonin use. 

Although recent medical advancements with the utility of neo-adjunctive chemotherapy have led to drastic improvements in the survival of patients with non-metastatic osteosarcoma, possible safer modalities of therapy that aim to slow its progression and improve therapeutic efficacy and minimize adverse side effects have remained as an unmet need among the scientific community. Therefore, in this systematic review, we delve into the possible utility of melatonin as a facilitating factor in therapeutic intervention and its positive effects on the quality of life in patients suffering from osteosarcoma.

## 2. Melatonin: The Basics

### 2.1. Synthesis and Secretion of Melatonin 

Melatonin (N-acetyl-5-methoxytryptamine) is a product produced rhythmically in response to changes in light perceived by the cells of the inner retina. This neuroendocrine agent is also synthesized and secreted peripherally, including the skin, bone marrow, gut, ovaries, and prostate [2,6,7]. Its regulation relies heavily on the master clockwork and neuronal stimulus of the suprachiasmatic nucleus [2,8,9]. This allows the temporal regulation of various biological processes and personalizes it to the organism’s inner processes and interaction with the external environment [7]. Melatonin production is mainly facilitated by the absence of light, in which its levels mainly peak in blood during the night at concentrations that vary between 80 and 120 pg/mL, while in the presence of light (during the day), its levels go between 10 and 20 pg/mL [2,3,7,8,9]. Studies have also reported high levels of melatonin in bile and CSF [2,3,7,8,9]. 

### 2.2. Childhood and Melatonin 

Interestingly, serum concentration of melatonin appears to vary with changes in the human body [2,7,10]. These concentrations continue to evolve with human development’s growth and inner changes. To illustrate, the immature central nervous system begins to produce melatonin in the neonatal period but fails to present the circadian rhythm. As the child matures, melatonin concentrations steadily increase, reaching a steady rhythm around the first year of life [10]. Melatonin synthesis continues to grow as the child ages and peaks around preschool [5,11]. These levels continue to decline steadily until puberty, where a minimal transient increase in melatonin levels is observed before it resumes decline [5,11]. This, as Gupta et al. propose, could be correlated to the growth of the skeletal frame of the individual [10].

### 2.3. Mechanism of Action and Physiology 

**Figure 1:**
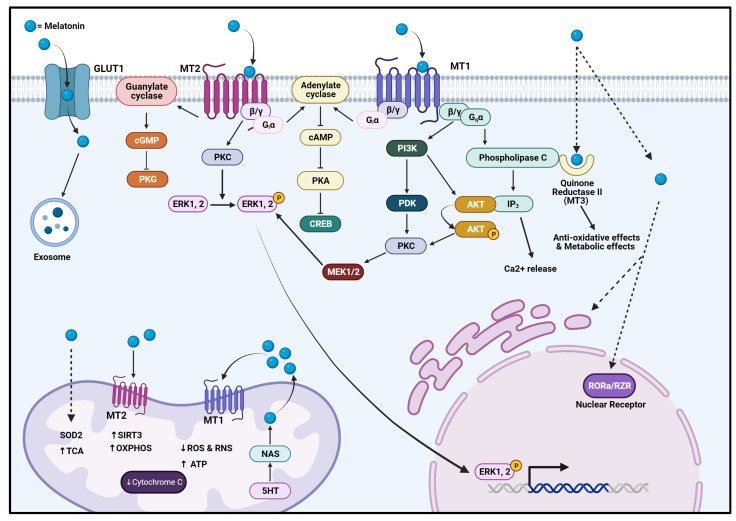
Mechanism of action of melatonin. The image was adapted and modified from [12,19,20]. Melatonin enters the cell through various receptors on cellular surfaces (such as MT1/2 and GLUT1 or passively diffuses into the cells and organelles. Melatonin utilizes receptors such as MT1/MT2, cytoplasmic receptor quinone reductase II, and nuclear receptor RORa/RZR, leading to various biological effects. It also contributes to the function and regulation of processes of other organelles, such as mitochondria, exosomes, and ER. AKT = protein kinase B, cGMP = guanosine 3’,5’-cyclic monophosphate, CREB = cAMP-response element binding protein, IP3 = inositol trisphosphate, MT1 = melatonin receptor 1, MT2 = melatonin receptor 2, OXPHOS = oxidative phosphorylation. PDK = pyruvate dehydrogenase kinase, PI3K = phosphoinositide 3 kinase, PKC = protein kinase C, PKG = protein kinase G, SIRT3 = sirtuin 3, TCA = tricarboxylic acid cycle, and SOD2 = superoxide dismutase 2. The dotted arrows indicate possible transmembrane translocation of melatonin molecules while the regular arrows indicate pathway activation and progression via pathway related molecules. The ‘closed’ lines indicate pathway inhibition. Created with BioRender.com.

Melatonin acts both centrally and peripherally. It can operate in receptor-dependent and receptor-independent fashions. It mainly acts via G-protein coupled melatonin receptors, MT1 and MT2, found throughout the body (Figure 1). These GPCR receptors can work independently utilizing their alpha subunit, leading to the activation of inhibitory G proteins, leading to activation of the PKC, ERK, and PI3K/Akt pathways with the addition of activating Gq proteins by dimerizing to MT2 receptors [12,13] (Figure 1). These receptors are found in different concentrations within the body, mainly throughout the central nervous system, cardiovascular system, immune system, skin, liver, kidneys, adrenal cortex, placenta, ovaries, and testes [13]. Melatonin can also compete with glucose and utilize GLUT1 receptors for cellular entry [14].

Moreover, its excellent lipophilic characteristics allow melatonin to act independently of receptors by freely moving between peripheral tissues, transversing the plasma membrane, and working directly on cellular proteins such as cytosolic receptors, mitochondrial proteins, and nuclear receptors and thus modulate signal transduction [7,15] (Figure 1). Melatonin’s function extends to facilitating exome formation and reducing endoplasmic reticular stress. The nuclear receptor RORa/RZR is utilized by melatonin to regulate gene transcription through the direct interaction with ROR response elements and affects 5-lipooxygenase as one of its target response genes [16,17] (Figure 1).

Overall, these functions indicate the critical role of melatonin in regulating signaling pathways mainly correlated to cell signaling, cell metabolism, and DNA damage response [12,14,18].

**Figure 2:**
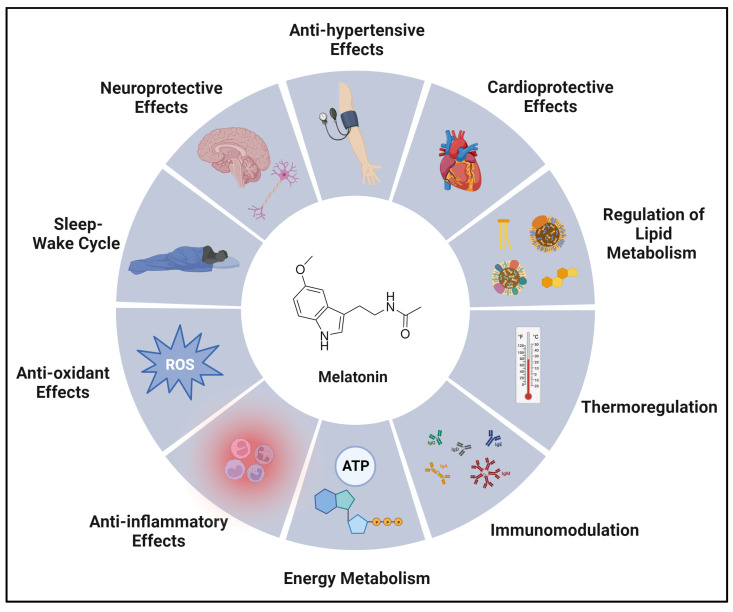
Biological functions of melatonin in various physiological sub-categories summarized in the illustration provided. ATP = adenosine triphosphate and ROS = reactive oxygen species. Created with BioRender.com.

Many studies have presented various contributions of melatonin in different aspects of biological functions. A simplified summary in Figure 2 and Table 1 below showcases melatonin’s multifaceted functions in biology. 

Table 1 below presents the promising findings on the effect of melatonin in various conditions.

### 2.4. Melatonin in Bone Metabolism and Remodeling

Melatonin contributes to bone repair and growth [4]. Melatonin plays a remarkable role in the modulation of MSC differentiation and bone formation [29]. Reports also shed light on its role in decreasing RANKL and macrophage colony-stimulating factor (M-CSF) mediated osteoclastogenesis and stimulation of osteoblastic activity, either through regulating gene expression or modulating osteoprotegerin activity, which has been linked to the MAPK and ERK signaling pathways [30,31]. Melatonin also suppresses osteoclastic activity through the inhibition of the transcription factor: the nuclear factor of activated T cell cytoplasmic 1 via the suppression of NF-κB activity [32]. A free-radical scavenger like melatonin also helps in protecting bone cells from oxidative stress and decreasing ROS secondary to osteoclastic activity [32]. 

Xiaofeng et al. have outstandingly presented the various evident effects of melatonin on osteogenic activity. The study supported melatonin’s role, suggesting that osteoblastic activity heavily relied on constant exposure to melatonin, where gene expression was correlated to circadian rhythm [4]. Furthermore, mesenchymal transformation mainly relies on interacting with these stem cells and the external stimulus. Melatonin increases concentrations of neuropeptide Y, which plays a role in MSC proliferation, migration, and osteogenic differentiation. It also modulates the ERK and Wnt pathways, which prevents osteoporosis secondary to estrogen deficiency, aging, and bone regeneration in fractures [4].

A summative illustration of the molecular modifications of melatonin on bone homeostasis has been presented in an exceptional systematic review by MacDonald and colleagues (2021), where the multifaceted effects of melatonin on bone pathologies such as osteoporosis and bone loss secondary to malignancy evident in preclinical and clinical settings is summarized [33].

Furthermore, the anti-inflammatory qualities of melatonin aid in other bone pathologies, including osteoarthritis, where the modulation of the SIRT1 pathway by reducing its activity leads to the reduction in NOS, PGE2, and cyclooxygenase 2 synthesis. All these advantageous effects remain that way based on the therapeutic doses administered. Increasing doses of melatonin up to 50 mg/kg had unexpectedly suppressed bone remodeling in mice [4,31,34,35]. Finally, the regulation of pro-inflammatory cytokines by melatonin in rheumatoid arthritis and osteoarthritis was secondary to alterations in clock gene expressions such as DEC2, CRY, and BMAL supporting the role of melatonin in synchronizing biological events such as bone health with circadian rhythm [33].

The bone remodeling cycle is a continuous balancing interaction between osteoblasts’ bone deposition and osteoclasts’ bone resorption that occurs in response to various interactions of biological processes with the external environment [36]. A balanced circadian rhythm and adequate sleep is essential for bone health [36]. Sleep is an essential factor in the bone remodeling cycle, where studies have presented a decrease in bone remodeling with an unchanged pace of bone resorption in states of inadequate sleep [34]. Preclinical studies aiming to explore the actions of sleep restriction on bone health in rats presented a significant decrease in osteoblast activity and osteoid formation in sleep-deprived rats [34,37,38]. Other findings showcased an unchanged bone resorption rate, with a peculiar decrease of osteoclast activity in sleep-deprived rats, contributing to the reduction in bone density facilitating osteopenia [34]. Since low levels of melatonin may affect normal sleep and wake cycles, this also may contribute to bone health and hence the correlation between melatonin levels, inadequate sleep, and bone health must be thoroughly investigated [36].

## 3. Osteosarcoma: Where Are We Now?

### 3.1. Osteosarcoma: The Facts 

Osteosarcoma, the second most common form of primary bone neoplasm, is primarily known for its vicious growth in a rapidly growing bone, which, timing-wise, would often be correlated to puberty [39]. It arises most commonly in the metaphyseal portion of long bones such as the tibia and femur and has a bimodal distribution of 10–14 years of age and 60 years old [39]. This aggressive tumor seeds itself and creates metastatic foci in other long bones and lung tissue and has a 27% 5-year survival rate [40]. Current treatment modalities include surgical interventions with polychemotherapy and adjunctive radiotherapy as secondary interventions. Recent research aims to explore the efficacy of immunotherapy in osteosarcoma. 

The pathogenesis of this malignancy presents a common pathophysiology, which includes abnormal signal transduction leading to uncontrolled cellular proliferation and protein translation, loss of apoptosis, angiogenesis, tissue invasion, and metastasis [9,39,41,42]. Environmental exposures, chromosomal abnormalities, changes in gene expression and regulation, and signal transduction anomalies lead to aberrant metabolism and growth factor production and, finally, cell cycle irregularities in the leading cellular players of tumorigenesis that include MSCs, preosteoblasts, and osteoblasts termed as cells-of-origin [41,43]. Common environmental exposures related to osteosarcoma include but are not limited to exposure to ionizing radiation, beryllium oxide, asbestos, and prosthetic use [39].

### 3.2. Cancer Cell Populations 

Studies have indicated the critical role of deregulation in the process of bone homeostasis in MSCs, correlating significantly to the formation of bone-originating neoplasms [44]. MSCs can be found throughout the body, and in the context of osteosarcoma, MSCs of various origins seem to be heavily recruited and promote tumorigenesis via the active interaction with components of a tumor-promoting microenvironment [39,44,45,46]. MSC can be recruited to the tumor site by TGFβ and cytokines such as SDF-1 and MIF. Recruited MSCs are then heavily influenced by factors such as INFγ, TNFα, IL-1α, and TGFβ to grow relentlessly, metastasize, and aid in the formation of malignancy-associated fibroblasts [41]. 

Furthermore, the MSC-derived osteosarcoma cancer stem cells (O-CSC) can influence and promote tumorigenesis through intercellular communication and utility of growth factor containing vesicles, which, as a result, enhance recruitment of circulating MSCs that will aid in tumorigenesis [47]. The varying surrounding microenviroment acts as a supportive factor in further OS-CSC heterogeneity, where the exploitation of bone signaling allows the formation of niches (as illustrated by Abarrategi and colleagues in 2016) as the perivascular niche, the endosteal niche, and the hypoxic niche. These O-CSC carry varying degrees of mutation burdens, aiding in creating further sub-populations that can adjust phenotypic expressions, survive in sacrospheres, and continue evolving in methods of survival [48].

### 3.3. Genomic Nature 

OS is known for its heterogeneity in genomic levels that vary from one patient to another and diversify in a single tumor [44]. Studies utilizing whole exome sequencing have presented a myriad of OS genomic defects, showcasing its complexity, diversity, and instability [44,49,50]. Whole genomic sequencing in pediatric patients in 2020 revealed that additional aberrant genomic modifications, such as chromosomal structural alterations secondary to chromothripsis, occurred in 20–89% of the patients, and 50–85% of patients have presented with kataegis that further promotes disease heterogeneity in OS [50]. This contributed to the formation of OS subclasses varying in morphology, metabolism, tumor microenvironment, immunogenicity, and metastatic potential, thus posing a challenge in evaluating the initial cause of osteosarcoma carcinogenesis [47]. Furthermore, deficiencies in DNA damage surveillance and repair also contributed to chromosomal structural abnormalities, deregulation of the tumor suppressor function, and uncontrolled cell cycle [41,43,51]. Recent studies focus on the stages of pathogenesis, where defects in the osteogenesis of MSCs predisposed to mutations lead to the formation of highly malignant OS colonies. 

As mentioned, common genetic alterations present as familial syndromes or sporadic mutations that predispose to OS are provided in Table 2 below. These defects affecting tumor suppressor genes, such as TP53 and retinoblastoma (Rb), are commonly associated with pediatric osteosarcoma cases. Overall, various genetic defects in TP53 were identified in 75–90% of OS cases [50]. Familial TP53 was located in 20% of OS cases [52]. Next generation sequencing has established that approximately 50–78% of OS cases have been associated with defects in Rb1 [50]. Sporadic cases of OS are attributed to the silent mutations affecting TP53 or Rb1 genes [41].

A plethora of genetic anomalies are correlated to the incidence of osteosarcoma, presented concisely by Chia-chin and colleagues in 2020 [50]. Such genetic alterations contribute to OS, but they are not necessarily found consistently in all OS patients. Some common genetic alterations identified were included in the Table 3 below:

Irregular expression of non-coding components of the genome, which include the various types of RNA involved in the epigenetic regulation of gene expression, have also been correlated to OS. These components play an essential role in various biological processes in which long non-coding RNA plays a role in cell cycle control, transcriptional regulation, cell growth, and development [41,62].

Table 4 showcases some of the different types of non-coding RNA expressed in higher quantities in osteosarcoma.

### 3.4. Signaling Pathways 

**Figure 3:**
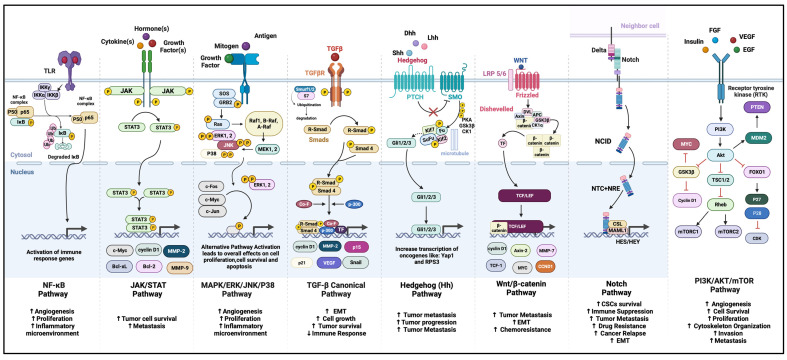
Signaling cascades involved in OS pathogenesis commonly such as the PI3K/Akt/mTOR, MAPK/ERK, TGFβ, Notch, Hedgehog, and NF-κB pathways have been evident in the different aspects of OS pathogenesis, summarized in the figure. These pathways can, independently or through cross-communication, aid osteosarcoma proliferation, survival, angiogenesis, migration, and invasion. Akt = protein kinase B, APC = adenomatous polyposis coli protein, Bcl2 = B-cell lymphoma 2, Bcl-xL = B-cell lymphoma-extra-large, CK1 = casein kinase 1, c-Myc = cellular myc, Co-F = co-factor, Dvl = dishevelled protein, EMT = epithelial mesenchymal transition, EGF = epidermal growth factor, ERK = extracellular signal-regulated kinase, FGF = fibroblast growth factor, GSK-3β = glycogen synthase kinase 3β, JNK = Jun N-terminal kinase, Kif = kinesin family member, MMP = matrix metallopeptidase, mTOR = mechanistic (formerly “mammalian”), NCID = NOTCH intracellular domain, NF-κB = nuclear factor kappa B, PI3K = phosphoinositide 3 kinase, R-SMAD = receptor-regulated SMAD, STAT = signal transducers and activators of transcription, target of rapamycin, SMAD = suppressor of mothers against decapentaplegic, SUFU = suppressor of fused homolog, TCF/LEF = T-cell factor/lymphoid enhancer factor, TGFβ = transforming growth factor-beta, TF = transcription factor, TSC 1/2 = tuberous sclerosis 1/2, VEGF = vascular endothelial growth factor, and WNT = wingless/integrated. Created with BioRender.com.

Abnormal expression of oncogenes and tumor suppressor genes secondary to changes in epigenetic and genetic elements leads to anomalies in cell biology, leading to aberrant signal transduction mechanisms and abnormal communication between various cellular processes and gene expressions. Many pathways are implicated in cancer cell formation, progression, survival, and metastasis in OS (Figure 3 and Table 5) [3,43,66,68,69,70,71,72,73,74,75,76,77,78,79,80,81,82,83,84].

Examples of signaling cascades involved in OS include those correlated to cell growth and proliferation, such as PI3K/Akt/mTOR, MAPK, and growth factor pathways. Studies have showcased significant gene mutations correlated to OS’s PI3K/Akt and MAPK/ERK pathways [41,85] (Figure 3 and Table 5). The JAK/STAT3 pathway also has a prominent role in OS proliferation, prevention of apoptosis, and immune regulation, where GFs (FGF, VGEF, etc.) and cytokines (IL-6, IL-10, IL-11, etc.) inappropriately activate STAT-3 protein and encourage OS progression [72,81,86]. Several other pathways (illustrated in Figure 3), such as the Hedgehog pathway, Wnt, SIRT1 and NOTCH signaling pathways, and TLR pathways, support tumor progression through the modulation of cell cycle progression and inhibiting cellular mechanisms of cell death and apoptosis. These pathways also aid in cells’ epithelial–mesenchymal transition (EMT), encouraging cancer cell migration, invasion, and metastasis. Hinton and colleagues explained the role of these factors in EMT in the pathophysiology of OS [87,88,89] (Figure 3 and Table 5).

Inter-cellular cross-communication via the Hedgehog pathway supports OS’s invasion and metastasis, supporting various oncogenic pathways in tumor progression and metastasis. Moreover, overexpression of Hes/Hey proteins involved downstream of the NOTCH signaling pathway was correlated to poor prognosis and survival [66] (Figure 3 and Table 5). The Wnt/β-catenin pathway also plays a pivotal role in OS-CSC renewal, embryogenesis, and tissue regeneration [70,74]. In the context of bone disease, this pathway affects the growth, survival, and differentiation of MSCs and interacts with different aspects of bone metabolism [74] (Figure 3 and Table 5).

**Table 5:** Signaling pathways involved in OS and their expression in OS.

Signaling Pathway Involved	Expression in OS	Function	References
SOX-9 via JNK Pathway	↑	↑ Tumor progression	[43,90,91]
Wnt Pathway	↑ (↑ in CSC)	↑ Cell proliferation ↑ Cancer cell survival ↑ Tumor metastasis ↑ EMT ↑ Chemoresistance	[43,70]
NOTCH Pathway	↑	↑ Tumor metastasis ↑ Drug resistance ↑ EMT ↑ Cancer relapse	[43,66]
Hedgehog Pathway	↑	↑ Tumor metastasis	[82]
SDF1-CXCR4 Pathway	↑	↑ MMP9 expression leading to invasion	[43]
PI3K/Akt/mTOR Pathway	↑	OS progression ↑ Angiogenesis ↑ Proliferation ↑ Invasion ↑ EMT ↑ Metastasis	[41,43,83,85]
JAK2/Stat3 Pathway	↑	↑ Tumor cell survival ↑ EMT ↑ Metastasis	[43,81]
RANK Pathway	↑	↑ Cancer cell migration ↑ Lung metastasis ↑ Pathological bone destruction	[43,51,81,92]
Hippo Pathway	↑	↑ Chemoresistance	[43,92]
ERK/MAPK Pathway	↑	↑ Angiogenesis ↑ Proliferation ↑ Inflammatory microenvironment	[43,69]
NF-κB Pathway	↑	Cancer proliferation Immune response	[52,93]
Fas/Fas ligand Pathway	↓	Caspase cascade Apoptosis regulation	[94]

Summary of the commonly studied signaling pathways involved in osteosarcoma, presented with their rate of expression and implicated effects. Akt = protein kinase B, EMT = epithelial mesenchymal transition, ERK = extracellular signal-regulated kinase, MAPK = mitogen-activated protein kinase, MMP9 = matrix Metallopeptidase 9, mTOR = mechanistic (formerly “mammalian”) target of rapamycin, NF-κB = nuclear factor kappa B, OS = osteosarcoma, PI3K = phosphoinositide 3 kinase, Wnt = wingless/integrated.

Besides the pathways mentioned, studies also implicate the RANK/RANKL/OPG pathway and exosomal/vesicle-based cellular communication in the initiation and progression of OS, proliferation EMT, invasion, and metastasis [45,56,66,94,95,96,97,98].

### 3.5. Cancer Cell Survival: Cytokines, Growth, and Transcription Factors 

Furthermore, osteosarcoma cells have also presented abilities to promote cellular proliferation via the secretion of autocrine growth factors such as insulin-like growth factor (IGF), transforming growth factor (TGF), and connective tissue growth factor (CTGF) [39,41,42]. Other factors, including VEGF, bone morphogenic proteins (BMPs), and hypoxia-inducible factor 1 (HIF1), also promote an optimal oncogenic microenvironment. Broadhead et al. presented in vitro studies that showcased a 30–50% decrease in OS with the inhibition of TGFβ [39]. Other studies showed that TGFβ played an essential role in EMT and was expressed in more significant affinities in high-grade OS [39]. Platelet-derived growth factor (PDGF) supported and preserved neoplasticity and cancer cell characteristics in OS [51].

PTHR1, the receptor primarily activated by PTH and PTH-rp, is a GPCR highly expressed in the kidney, cartilage, and bone. Abnormal expression of this receptor has been correlated to incidence and malignant transformation of OS. It modulates several signaling pathways, such as MMP signaling, ECM modulation, osteoblastic differentiation and proliferation, and others involved in bone metabolism [99]. Cell studies revealed that parathyroid hormone (PTH) and parathyroid-related peptide (PTH-rp) supported the migration of OS cells (MG-63 and SaOS-2), downregulated pro-apoptotic mechanisms, and aided in chemoresistance [39]. Receptor parathyroid receptor-1 (PTHR1) expression in OS cells is a poor prognostic marker [99].

Aberrant expression of transcription factors aids in the mutation load, leading to uncontrolled and aggressive neoplasm proliferation. The transcription factor c-Myc has been studied extensively in osteosarcoma and promoted cell growth and proliferation [60]. Han and colleagues have noted the role of c-Myc in invasion via the MEK-ERK pathway in OS [60]. In addition, the transcription factor nuclear factor κB is highly expressed in OS tissue and contributes prominently to various stages of cancer progression. A downregulation of PTEN expression in the OS tissues as a secondary effect related to the increase in levels of NF-κB [52,79]. Overall, these factors and higher levels are often correlated to higher grades of osteosarcoma [39,41,42,51,52].

Cytokines have been a significant influence on tumor viability (Table 6). Growing neoplasms take advantage of specific cytokines and facilitate pro-inflammatory conditions that promote migration, invasion, and metastasis. The interconnective communication between these various cytokines with diverse sets of cells contributes to optimizing the tumor microenvironment [52,100,101].

### 3.6. Microenvironment, Angiogenesis, and Tumor Metabolism 

Optimal blood supply aids in developing cancer colonies, survival, and viability. Intraosseous neoplasms such as OS aim to take advantage of the osteolytic destruction of surrounding tissue secondary to cancer biology and, often in an autocrine/paracrine fashion, promote angiogenesis via utilizing hypoxia-inducible factor 1α (HIF1α). Zhao and colleagues have deduced the significance of HIF1 in OS, in which elevated HIFα expressions were identified in 56.82% of OS cells [103]. The overexpression of HIF1α contributes to activating the vascular endothelial growth factor (VEGF), a vital factor that promotes angiogenesis. The excess production of both factors facilitates immature vascular supply to tumor colonies that further support its survival [65,104].

Cancer cells require high energy sources to thrive, which is commonly attributed to the Warburg phenomenon. The rate of aerobic glycolysis (Warburg effect) and subsequent lactate production was found to be increased in MSCs, a phenomenon triggered by neighboring/adjacent OS cells [105,106]. The MSCs, in turn, via the MCT1 transporter (reverse Warburg effect), feed the osteosarcoma cells with the lactate, which is then converted to pyruvate to enter the Krebs cycle [105,106]. In an in vivo mice model of osteosarcoma, elevation in lactate levels and lactate shuttling via increased expression of MCT1/4 was observed [106]. This, in turn, resulted in an increase in oxidative phosphorylation and ATP production in OS in the presence of MSCs and accumulation of lactate in the OS cells, thereby enhancing their metabolism and allowing the cancer cells’ aberrant proliferation, growth, and metastasis [106]. The relationship between this proposed metabolic state in OS and the classic Warburg effect in many neoplasms has not been studied. In addition, the role of melatonin in this phenomenon has not been explored, but itis a promising angle to explore in osteosarcoma.

### 3.7. Cancer Resistance 

Standard therapeutics for the treatment and management of OS include surgical excision of the tumor with adjuvant pre/post-operative polychemotherapy that uses one or more chemotherapeutic drugs such as methotrexate, doxorubicin, cisplatin, and ifosfamide. Additionally, other treatment options include radiotherapy in conjunction with chemotherapeutic intervention. This regimen can be personalized based on the location, tumor burden, patient demographics, and metastasis status of the malignancy [42]. Novel treatments include bisphosphonate use and immune modulation strategies [94].

**Figure 4:**
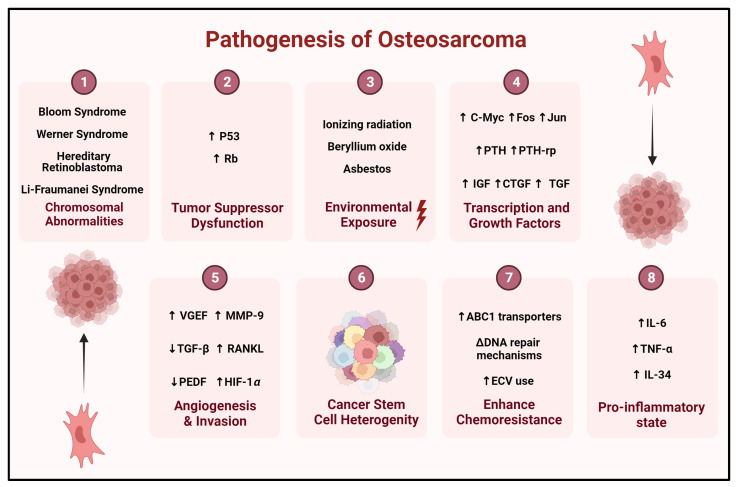
Simplified summary of the pathogenesis of OS. CTGF = connective tissue growth factor, ECV = extracellular vesicles, HIF-1α = hypoxia-inducible factor 1 subunit alpha, IGF = insulin growth factor, MMP-9 = matrix metallopeptidase-9, PEDF = pigment epithelium-derived factor, PTH = parathyroid hormone, PTH-rp = parathyroid-related peptide, RANKL = receptor activator of nuclear factor kappa beta, TGF = transforming growth factor, TGFβ = transforming growth factor-beta, VEGF = vascular endothelial growth factor. Created with BioRender.com.

Osteosarcomas, via inherent capabilities or acquired mechanisms, develop resistance to drug interventions and become less responsive to therapeutic intervention. Resistance to current management programs is multifactorial and is primarily attributed to the heterogeneous nature of O-CSCs. The heterogeneity and diversity in the origin of the OS cells, the tumor microenvironment, and gene mutations help the tumor achieve resistance to current anti-tumorigenic therapeutics, enhancing their survival against hypoxia and supporting immune evasion while retaining and evolving self-renewal mechanisms [107]. Furthermore, the upregulation of ABC transporter, P-gp, and MDR proteins and the presence of cancer stem cells have been implicated in the development of resistance in OS and are characterized by the poor response to various chemotherapeutic drugs [63,94,95,96,107,108]. Other resistance mechanisms investigated in high-grade OS involve the upregulation of ABC1 transporters, changes to the expression of modified DNA repair mechanisms, and the use of extracellular vesicles via modulating microRNAs with the surrounding cells [95,96]. Figure 4 summarizes the various effectors in the pathogenesis of OS.

## 4. Melatonin in OS

### 4.1. Melatonin in Malignancy 

**Figure 5:**
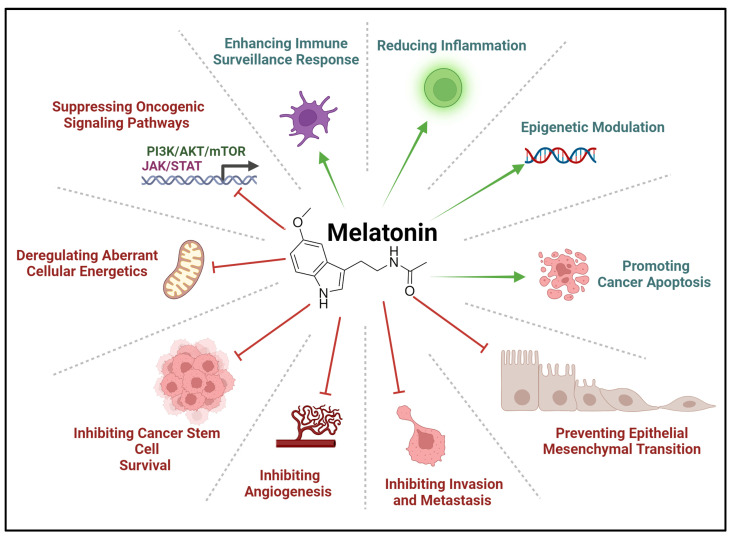
Effects of melatonin in cancer. These various oncostatic effects are found through preclinical and clinical studies in different cancers. Created with BioRender.com.

The oncostatic characteristics of melatonin have been explored in various types of malignancy. It was evident that shifts in the sleep-wake cycle had significantly enhanced the risk of genomic instability, contributing to tumorigenesis [109,110,111]. Animal models undergoing pinealectomy have consistently illustrated an increased tumor burden, although two studies reported the opposite effect [68,112]. A myriad of scientific experiments revealed melatonin’s oncostatic qualities in diverse neoplasms. In vitro and in vivo studies complimented this hypothesis, which encouraged the proposition of the use of melatonin in the treatment of cancer [113,114,115,116,117,118]. Melatonin has exhibited anti-tumorigenic properties in multiple neoplasms (Table 7 and Figure 5). These were mainly explored in in vitro and animal studies, where melatonin affects various aspects of cancer cell biology:

The myriad of anti-cancer properties of melatonin encourages ongoing and future research to develop melatonin as a potent influence on future cancer therapy (Table 7 and Figure 5). In particular, the nuanced relationship between skeletal expansion during puberty and the stark decline in melatonin synthesis was deduced to contribute to the pathogenesis of osteosarcoma among this group of the population [3,5,39]. Epidemiologic studies correlated low levels of melatonin to OS patients. This, and the proposed role of melatonin in bone metabolism, urges scientists and researchers working in this area to focus on melatonin’s prospective oncostatic roles, particularly in primary bone malignancy. 

### 4.2. Melatonin, the Cell Cycle, and Cell Signaling

Melatonin exerts oncostatic effects via the modulation of cell cycle activity. Melatonin can inhibit different components of cell cycle control. These could either inhibit the cell cycle-related proteins such as PKC and cyclic-dependent kinases (CDK), decrease cyclin D proteins, increase CDK inhibitors, or inhibit prominent pathways involved in the cell cycle [126]. Melatonin was also evident in modulating telomerase activity and regulating cell autophagy [127]. As showcased in Table 8, melatonin interferes in MG-63 cell proliferation via the decrease in CDK1, CDK4, cyclin B1, and cyclin D1 leading to the accumulation of cells in the G0/G1 phase of the cell cycle, blocking progression through G2/S/M phases in variable concentrations. 

**Figure 6:**
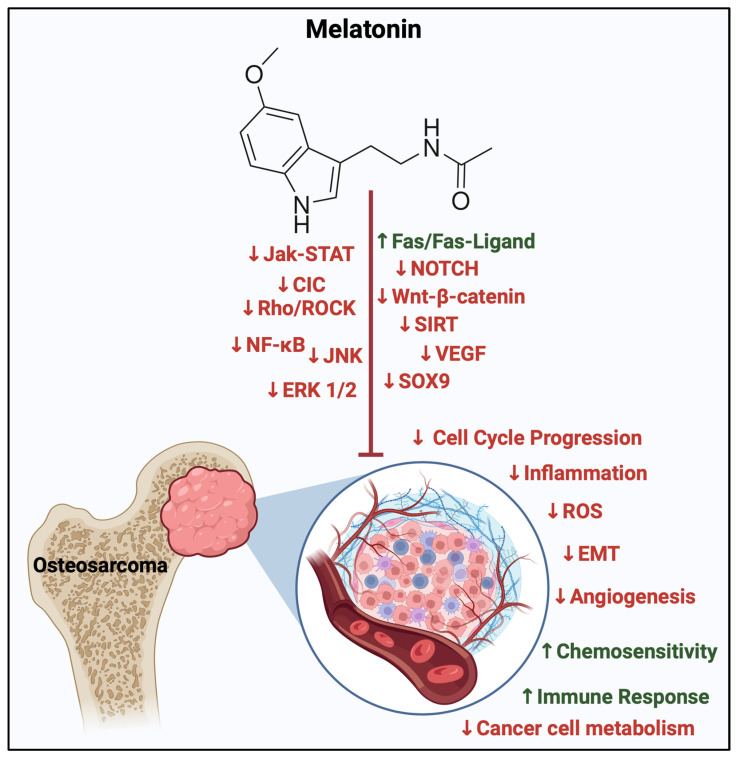
Oncostatic effects of melatonin in osteosarcoma. An illustrative summary of inhibitory (red) and enhanced (green) effects of melatonin. These include inhibiting cell cycle progression signaling pathways involved in OS tumorigenesis, such as SIRT, JAK-STAT, Rho/ROCK, ERK1/2, JNK, NOTCH, and Wnt-catenin. Melatonin also induces apoptosis through interactions with Fas/Fas-ligand, modifies cancer metabolism and immune response to malignancy, and modifies inflammatory conditions of the surrounding microenvironment by reducing ROS and inflammation. Finally, it enhances the sensitivity of tumors to current chemotherapies. CIC = capicua transcriptional repressor, EMT = epithelial-mesenchymal transition, ERK1/2 = extracellular signal-regulated kinase, NF-κB = nuclear factor kappa B, Rh0/ROCK = Rho-associated protein kinase, ROS = reactive oxygen species, and VEGF = vascular endothelial growth factor. Created with BioRender.com.

Convincing proof indicates that melatonin exhibits both cytotoxic and anti-metastatic effects in diverse cancer cells, with these actions appearing to be specific to different cell types, including human osteosarcoma cell lines [62,115,118,119,120,128,129,130,131]. The role of melatonin in signaling cascades involved in the different stages of cellular proliferation and stability has been investigated extensively (Table 8 and Figure 6). 

Noteworthy pathways pivotal in osteosarcoma advancement that have been evaluated in the presence of exogenous melatonin include the MAPK signaling pathway family. These intercommunicating pathways that include the c-Jun N-terminal kinase (JNK) and extracellular signal-regulated kinase (ERK) pathway that aid in cell proliferation and progression, as stated in Section 3.4. Variable mmol/L increments of melatonin were used, where initially there were no changes in 24 h of exposure, but there were changes in cell activity such as motility in 48 h, with changes in phosphorylation of components of the MAPK, ERK and JNK pathways in the U2OS and HOS cell lines [132].

Moreover, the Wnt/β-catenin signaling network was also assessed in different osteosarcoma cell lines (Saos-2, MG63, and U2OS) where a dose-dependent decrease in cell survival, migration, and metastasis was seen more prominently at 1 mM of melatonin [62].

The process of epithelial–mesenchymal transformation creates an aggressive and more invasive OS niche. What is seen is that through inhibiting SOX-9 mediated signaling, melatonin impairs the formation of osteosarcoma sacrospheres and EMT initiation and progression, thereby decreasing OS invasion. These effects were significant in cell and animal studies [133]. Likewise, the inhibition of the SIRT1 pathway in micromolar concentrations of melatonin contributed to an increase in the apoptosis index, with an overall reduction in cell growth [134].

Finally, the Rho/ROCK pathway, commonly known for its transduction of inhibitory signals, was recently correlated to play a pivotal role in vasculogenic mimicry in osteosarcoma cells [135]. This pathway, along with others aiding in OS metastasis, has been explored by Zhang and colleagues (2021) through cell and in vivo models, where 1 mM of melatonin impeded mitochondrial activity of OS cells and inhibited cell-in-cell structural formation, utilizing 3D printed magnesium–polycaprolactone as a delivery agent [136]. 

Table 8 below has been provided as a summative representation of the preclinical trials in preclinical osteosarcoma studies. Based on the suggested data, the conclusion deduces that melatonin has been effective as an oncostatic molecule in pharmacological concentrations ranging from micromolar to millimolar.

**Table 8:** Melatonin and OS-related preclinical studies, signaling cascades, and their effects.

Signaling Cascade	Study Model	Type of Model	Melatonin Concentration	Effects	References
Cell Cycle Modulation	Cell Study	MG-63 cells	4–10 mM	↑ Cells in G0/G1 phase ↓ G1 phase progression via (↓ cyclin D1 and CDK4) ↓ G2/M phases progression via (↓ cyclin B1 and CDK1)	[137]
Cell Cycle Modulation	Cell Study	MG-63 cells	0–0.1 M	↓ G0/G1 cell cycle phase at 9 mM	[138]
SOX9 suppression EMT Pathway	Cell Study	HOS, MG-63, and U2	1 mM	↓ Migration ↓ Invasion ↓ Sarcosphere formation OS-CSC ↓ EMT markers	[133]
	Animal Study	Mice	100 mg/kg	↓ Initiation and metastasis	
JNK/ERK Pathway	Cell Study	U2OS, HOS	0.25, 0.5, 1.0, 2.0 mmol/L	No effect of melatonin after 24 h exposure ↓ Cell motility after 48 h ↑ Phosphorylation of ERK1/2 ↓ Phosphorylation of JNK1/2 in U2OS and HOS cells	[132]
JPX-Wnt/β-catenin	Cell Study	Saos-2, MG63, and U2OS	0.1, 0.5, 1, 1.5, and 2 mM	↓ Cell survival rate ↓ Cell viability in a concentration-dependent manner (more evident from 1, 1.5, 2), dose-dependent ↓ Migration ↓ Metastasis	[62]
SIRT1 inhibition	Cell Study	9607	250 µM 500 µM 1000 µM	↓ Cell growth ↑ Apoptotic index ↓ Adhesion ability ↓ Migration ability ↓ Glutathione (GSH) levels	[134]
miR-424-5p/VEGFA	Cell Study	SaOS-2 and MG-63 cells	1–1000 μM	↓ Cell viability beyond 50 μM ↓ VEGFA mRNA ↓ Protein expression ↓ Secreted levels of VEGFA ↑ miR-424-5p expression in microenvironment.	[65]
CIC, Rho/ROCK cAMP/PKA	Cell Study	U2OS, 143b, hFOB1.1, MG63, HOS, OS patient tissue samples, VX2	1 mM	↓ OS development ↓ CIC activity ↓ Mitochondrial biogenesis ↓ Mitochondrial function	[136]
	Animal Study	Rabbit			

Concise list of the preclinical trials on the effects of melatonin on osteosarcoma cells, showcased with affected cellular pathway, type of study, type of cell/animal, melatonin concentration used, and denoted results. CDK = cyclin-dependent kinase, ERK = extracellular signal-regulated kinase, OS-CSC = osteosarcoma–cancer stem cell, EMT = epithelial–mesenchymal transition, SIRT1 = sirtuin (silent mating type information regulation 2 homolog) 1, VEGF = vascular endothelial growth factor, JNK = Jun N-terminal kinase, and Wnt = wingless/integrated.

### 4.3. Tumor Microenvironment, Immune Response, and Oxidative Stress

Tumor microenvironment is a biosphere of various cell types and blood vessels that support tumorigenesis. In osteosarcoma, the TME consists mainly of a similar setting, with additional bone cells, and vascular and stromal cells in the mineralized extracellular matrix. The intercommunication between normal bone metabolism and surrounding immune cells through cytokines and growth factors is exploited in the TME [139]. Thus, osteosarcoma cells result in aberrant immune cell differentiation and recruitment to optimize cancer-thriving conditions [140].

Melatonin has succeeded in showcasing a well-studied relationship with the immune system, as suggested in Table 1. Preclinical studies showcased how melatonin has bidirectional communication with the immune system utilizing common endogenous substances, including ACTH, acetylcholine, growth hormones, somatostatin, endorphin, and vasoactive intestinal peptide [15,28]. The heavy distribution of melatonin receptors in immune cells supports its role in enhancing innate and acquired immunity [7,8,27,28,93]. 

These characteristics allow it to moderate immune and inflammatory responses in tumor microenvironments with responses to surrounding cells. This is achieved by regulating the expression of various pro-inflammatory mediators such as IL-6, TNFα, and IFN γ. Vital immune cells in TME include tumor-associated macrophages, NK cells, Treg cells, and cytotoxic T cells. The abundance of melatonin receptors on various cells of immune cells facilitates the activation, differentiation, recruitment, and survival of immune cells, such as T cells, in the presence of melatonin. Melatonin simultaneously aids in reducing such drastic inflammation and oxidative stress in surrounding tissue by decreasing IL-2, TNFα, IFN-γ, and COX-2 and improving IL-4, IL-10, and IL-27 expression [15].

Furthermore, melatonin creates a cancer-hostile environment by targeting tumor cells and creating a TME with elevated ROS levels, leading to oxidative damage and, eventually, cancer cell death, as reported in a hepatocellular cancer cell study by Dominguez (2016) and colleagues [141]. This has not been studied extensively in OS but could be a potential area to explore.

Primary bone tumors, such as osteosarcoma, can create a hostile ROS-rich environment as it progresses [142]. The cytoprotective features of melatonin against oxidative stress allow it to act directly and scavenge free radicals via its aromatic indole ring that acts as an electron donor, regulating antioxidative enzymes. It also stimulates antioxidant synthesis, decreases free radical production, and reduces the production and activity of reactive oxygen species [7,123]. Melatonin’s direct targeting of the NF-κB signaling pathway leads to a secondary reduction of free radicals [76]. This was evident through the reduction of ROS generation in 143B cells in 100 μM of melatonin [3]. Also, melatonin has showcased conditional pro-oxidant effects in elevated (millimolar) concentrations that lead to Fas-induced apoptosis, mitochondrial membrane instability, and mitochondrial complex III binding [143,144,145,146,147,148,149,150]. 

### 4.4. Melatonin and Cancer Metabolism

Aberrant utilization of glucose via aerobic glycolysis and inhibition of mitochondrial-related metabolism, known as the Warburg effect, has been a key finding in cancer cell biology [151,152]. It enhances tumorigenesis by amplifying energy sources and promoting cell proliferation [151,153]. In osteosarcoma, the reprogramming of metabolic activity aided its progression making it more aggressive and was reported to even enhance drug resistance [154]. Common metabolic alterations involve glycolysis, amino acid, lipid production, and the TCA cycle [155].

Moreover, research exploring the metabolic changes in cancer has also showcased drastic cancer cell metabolism in different phases of the day, in which glycolysis is more prominent during the day, where cancer cells depend on oxidative phosphorylation during the latter half of the day [130]. 

In addition, melatonin counteracts the Warburg effect and stops the related metabolic processes by directly or indirectly suppressing HIF-1α. HIF-1α is stabilized with the help of reactive oxygen species created in more significant quantities in hypoxic environments [129]. Melatonin also participates in various metabolic pathways, including aerobic glycolysis, gluconeogenesis, the TCA cycle, and the pentose phosphate pathway [129,130]. Melatonin regulates the various signaling pathways involved in these metabolic cascades or directly inhibits glucose transportation (GLUT) and enzymes (G6PDH activity) participating in metabolism [130]. This was evident in tumor cell studies supplemented with melatonin. Other preclinical studies have also noted decreased linoleic acid (LA) uptake, LA metabolism, and fatty acid metabolism in mice with hepatomas in the presence of melatonin [156]. 

Finally, these functions of melatonin, although not centered on OS, the overlapping rewiring of the metabolic processes among the different cancers including OS can be used to good advantage to further evaluate the effect of melatonin in OS with regards to its effect on aberrant metabolic alterations that occur in OS. However, the previously mentioned “reverse Warburg” phenomenon mentioned in OS has not been extensively studied, and the role of melatonin in the phenomenon is unclear and remains another area of investigation that needs to be addressed [105]. Similarly, the association between metabolic status shifts in day–night settings, mentioned previously, could aid in learning more about the undetermined importance of melatonin in cancer metabolism.

### 4.5. Melatonin and Synergistic Effects with Therapeutics 

The oncostatic effects of melatonin have been highly evident from past and ongoing research, but the implementation of melatonin in cancer treatment continues to be scrutinized. Its utility as an independent regimen remains unclear. However, many studies have studied the efficacy of co-administering melatonin in current management programs (Table 9). Using melatonin in combination with other standard chemotherapeutic drugs can either be beneficial by decreasing the side effects of current treatments or by enhancing their oncostatic effects [157,158,159]. For example, supplementing melatonin with chemotherapeutics has been investigated in patients with solid tumors, including breast cancer and lung cancer neoplasms of the GI tract and head and neck. Melatonin combination therapy reportedly improved chemotherapeutic sensitivity and other specific parameters such as myelo-immune suppression, neurological and cardiac toxicities, thrombocytopenia, and stomatitis [157,158]. Hrushesky (2021) and colleagues have showcased improved survival rates in patients taking 20 mg melatonin in the evening with their etoposide/cisplatin therapy [160].

Supplemental melatonin was also assessed for its role in aiding patients with general symptoms of advanced cancer. It was using the role of melatonin in regulating the sleep–wake cycle to one’s circadian rhythm. Melatonin improved fatigue in breast cancer patients, while other studies have reported the improvement of sleep and decreased delirium with melatonin supplementation in advanced cancer patients [164]. 

For osteosarcoma, co-administration of melatonin with standard chemotherapeutics has showcased various results in varying melatonin concentrations, as provided in the table below [163] (Table 9). Another mode of delivery of melatonin was evaluated, where melatonin/HPβCD scaffolds were utilized to optimize delivery that enhances the apoptotic effects of melatonin, which could be deduced to be secondary to an improvement in half-life [138]. This suggests an alternative administration system for melatonin administration to consider in OS therapy. 

## 5. Melatonin in the Clinical Setting 

### Clinical Studies on Melatonin’s Effects on OS: Prospects and Limitations

There is an abundance of cell and animal studies that prove the oncostatic effects of melatonin in various malignancies in clinical trials. Nevertheless, an endeavor to study the anti-cancer effects of melatonin in OS patients in a clinical setting appears to be minimal. Utilizing search words such as “Osteosarcoma” or “Bone Cancer” or “Bone Neoplasm” and “Melatonin” yielded no results on ClinicalTrails.gov (accessed on 8 December 2023). A similar search performed for melatonin as an intervention for “Cancer” generally has resulted in 46 clinical trials (accessed on 8 December 2023). Eleven of the forty-six trials (23.9%) of the studies presented results, while 35/46 (76.08%) did not report any results on the website. 

An additional component to reflect on is the limiting nature of integrating preclinical data in a clinical setting. Reports suggest that factors such as (1) differences in the circadian rhythms (known to influence cancer incidence and progression), (2) differences in metabolic rates (that should affect the response to anti-cancer drug interventions), (3) immune responses, and (4) genetic variations since humans are diurnal when compared to most experimental animals (rats or mice), known to be nocturnal, must be carefully considered in melatonin-related research [165,166,167,168,169].

While being diurnal or nocturnal may not affect melatonin synthesis, the fact that this may affect susceptibility to the disease, expression of genes and proteins, immune responses, and drug metabolism must be factored in to determine optimal dosage and the timing and frequency of melatonin intervention while translating melatonin-related anti-cancer effects from bench to bedside [165,166,167,168,169].

Moreover, the accurate pharmacokinetics of melatonin supplementation remains uncertain, which leads to a lack of consensus on a single therapeutic dose. Several attempts were made to administer melatonin using various methods that showcased variable bioavailability and half-lives, yet a clear conclusion was not exhibited [123]. Varying dosages have been proposed for multiple cancer studies. This can be highlighted by comparing the variable effective dosages presented in multiple preclinical trials; these were then extrapolated to propose a suitable dosage for 60 kg adults. These had presented heterogeneity in suggested dosages ranging from 1 to 195 mg. RCTs on the utility of melatonin adjunctively in breast cancer patients have also revealed that several studies implement low dosages, such as 3 mg/day and 10–20 mg/day, that have showcased improved 1-year survival rates [45]. 

Furthermore, after optimizing dosage in OS, another factor to consider would be to study the optimal duration, follow-up method, potential toxicities, and side effects [170]. Another suggestion for future research is reflecting on the heterogenicity of a patient’s circadian cycle, its effect on tumors, and its consideration in therapeutic administration timings [169]. Thus, the lack of standardized protocols for melatonin administration in osteosarcoma clinical trials can complicate data interpretation across different studies. Consistency in study design and methodology is essential for drawing meaningful conclusions. In addition, some gaps that can be further explored would be the role of melatonin in the variable grading and genetic morphology of OS. Further research should explore melatonin’s oncostatic potential, particularly on the RANKL/RANK/OPG axis, PTHR expression in OS, and its possible role in the “Reverse Warburg Effect”.

This sheds light on the dire need for future scientific endeavors to implement preclinical findings and consistent experimentation of melatonin’s use in pediatric osteosarcoma, where repetitive inquiries around the optimal therapeutic dosage, administrative method, and correlation to the body’s physiological rhythmic secretion of melatonin. 

## 6. Conclusions and Future Perspectives 

In conclusion, current evidence showcases promising discoveries and notable limitations in the area of melatonin research with respect to its effect in the treatment of osteosarcoma. Several studies have showcased the complexity behind the pathogenesis of osteosarcoma and the pharmacokinetics of exogenous melatonin use. These investigations have delved into potential synergies between melatonin and conventional chemotherapies, utilizing delivery systems like micro/nanoparticles and inclusion complexes to augment its effectiveness. However, there remain challenges in translating preclinical evidence into clinical settings. The lack of standardized protocols, study design, optimal administration modality, patient heterogeneity and a large body of evidence on the role of the timing of melatonin administrations linked to its robust role in the circadian rhythm in melatonin warrants more focused research in this regard to understand the oncostatic efficacy of melatonin in OS. Future research incentives could address such elements to advance our understanding and harness the therapeutic benefits of melatonin.

## Figures and Tables

**Table 1:** Biological effects of melatonin.

Pathology	Effects of Melatonin	References
Lipid Metabolism	↓ Dyslipidemia by ↓ levels of triglycerides and ↓ total cholesterol. Exogenous melatonin has secondary effects in ↓ Waist circumference ↓ BMI	[21,22]
Reproductive Health	↓ GnRH release ↓ LH levels. ↓ Melatonin was associated with hypothalamic amenorrhea and precocious puberty.	[1]
Cardiovascular	↓ Blood pressure	[7,23]
Energy Metabolism	↑ Glucose tolerance ↑ Insulin sensitivity ↓ Body weight	[24]
Thermoregulation	↓ Body temperature	[1]
Neurodegenerative Diseases and Dementia	↑ Cognitive thinking ↑ Neurogenesis ↑ Anti-aging effects	[1,25,26]
Stroke	↓ Infarction volume	[7]
Psychiatric Conditions	↓ Melatonin in depression, anxiety, schizophrenia, and autism	[26]
Immunity	↑ Anti-inflammatory cytokines ↓ Proinflammatory cytokine production (COX and iNOS)	[27,28]

BMI = body mass index, COX = cyclooxygenase, GnRH = gonadotropin hormone-releasing hormone, LH = luteinizing hormone, and iNOS = inducible nitric oxide synthase.

**Table 2:** Familial syndromes predisposing to OS.

Familial Syndrome	Gene	Inheritance Pattern	Function	References
Li–Fraumeni	p53	AD	↓ Cell growth ↓ Differentiation ↑ Cell apoptosis	[41,46,52,53,54]
Retinoblastoma	RB1	AD	Cell cycle regulation	[43,46,54]
Bloom	BLM	AR	DNA helicase	[54]
Werner	WRN	AR	DNA helicase	[54]
Rothmund Thompson	RECQL4	AR	RecQ helicase	[54]

Familial syndromes predisposing to osteosarcoma, presented with affected gene, inheritance patterns, and function of the gene. AD = autosomal dominant, AR = autosomal recessive, BLM = bloom syndrome protein, RB1 = retinoblastoma 1, RecQ = recombination Q helicases, and WRN = Werner protein.

**Table 3:** Genetic alterations implicated with OS can either be overexpressed or inhibited in OS initiation, progression, and viability.

Gene	Expression in OS	Function	References
PTEN	↓	↓ Cell growth ↓ Differentiation ↑ Cell apoptosis	[52]
WWOX	↓	Tumor suppressor gene	[41,53,55]
SOX5	↑	↑ Transcription factor synthesis ↑EMT, invasion, and migration	[41]
CDKN2A	↓	Cyclin-dependant kinase	[41,56]
INK4A	↓	CDK4 Cell cycle	[57,58]
MDM2	↑	Oncogene	[41,59]
c-Myc	↑	Oncogene	[41,60]
C-fos	↑	Protooncogene	[61]
RUNX2	↑	Oncogenesis	[41,55]

Genetic modifications correlated to osteosarcoma, presented with their studies’ expression pattern in OS and their function in OS pathogenesis. c-Myc = cellular myc, CDKN2A = cyclin-dependent kinase inhibitor 2A, EMT = epithelial-mesenchymal transition, INK4A = CDK inhibitors superfamily, MDM2 = murine double minute 2, PTEN = phosphatase and tensin homolog, RUNX2 = runt-related transcription factor 2, SOX5 = S RY-Box transcription factor 5, WWOX = WW domain-containing oxidoreductase.

**Table 4:** Non-coding RNA highly expressed in OS and their various effects on OS biology.

Non-Coding RNA Type	Non-Coding RNA	Effect	References
miR	miR-101, miR-574–3P, mi-R-20a, mi-R-19a, miR-16, miR-140, miR-150, miR-29, miR-133a, miR26a, miR-29b-1, miR-200b, miR-181, miR-205, miR-424, miR-106, miR-519	CSC formation and proliferation Activation of PI3K/Akt pathway Activation of JAK/STAT pathway Cell invasion ↓ IL-2 Lung metastasis	[41,63,64,65,66]
lncRNA	MALAT1, HOXD-AS1, TUG-1, LINC00161, SNHG16RO, NEAT1, SARCC, MIR17HG, OIP5, FENDRR	Tumor initiation Tumor proliferation Migration Invasion	
circRNA	hsa_circRNA_103801, hsa-miR-370-3p, hsa-miR-338-3p, hsa-miR-877-3p, CircTCF25, CircMMP9, Circ001621, CircEPSTI1, Circ0001658, Circ-LARP4	Cell proliferation Hinder cell death Tumor invasion	[41,67]

Role of various types of non-coding RNAs in the different stages of osteosarcoma biology. circRNA = circular RNA, CSC = cancer Stem Cell, lncRNA = long non-coding RNA, miR = microRNA, and RNA = ribonucleic acid.

**Table 6:** Cytokines involved in the various aspects of tumor biology.

Cytokine	Effect	References
IL-6	↑ Glycolytic metabolism in OS cells ↑ Lung metastasis ↑ MEK/ERK1/2/hypoxia-inducible transcription factor-1α (HIF-1α)	[52,102]
TNFα	↑ Undifferentiated cells ↑ Neo-angiogenesis ↑ M2 macrophage recruitment	[52,100,101]
IL-34	↑ Neo-angiogenesis ↑ M2 macrophage recruitment	[52,101]

Summary of the most common cytokines involved in tumorigenesis and osteosarcoma formation, presented with known effects. OS = osteosarcoma and IL-6/34 = interleukin 6/34.

**Table 7:** Melatonin’s effects in different neoplasms.

Malignancy	Effects	References
Neuron Malignancy	↓ Neuroblastoma via ↓ VGEF	[113,119]
Breast Cancer	↓ Risk of breast cancer ↓ CDK2, CDK4 ↓ IGFR ↓ HIF-1α ↓ VEGF ↑ miR-152-3p	[45,114,115,116,117,120]
Ovarian	↓ Oxidative stress ↓ CDK2, CDK4 ↓ Risk of ovarian cancer ↓ Akt/ERK/JNK pathway ↓ NF-κB pathway	[114,116,118]
Lymphoproliferative Pathologies	Cell cycle arrest ↓ Bcl-2 Mitochondrial membrane depolarization Cytochrome c release activation of caspase-3 in lymphoproliferative disease.	[9,114]
Lung Cancer	↑ Cancer cell migration and variability 2/2 ↑ JNK/MAPK pathway (6)	[114,121]
Renal Cancer	↑ Bim → ↑ apoptosis	[122]
Gastric Cancer	↓ RZR/RORγ → ↓ angiogenesis	[97]
Colon Cancer	↑ Apoptosis ↓ TGF	[114,123]
Prostate	↓ Cell growth of both androgen-dependent and androgen-independent prostate cancer ↑ miRNA3195 and miRNA374b	[124,125]

A summary of some of the effects of melatonin against cancer, as studied and published on various types of malignancies, provided the illustrated effects. Akt = protein kinase B, CDK2/4 = cyclin-dependent kinase 2/4, Bcl-2 = B-cell lymphoma 2, Bim = Bcl2-like protein 11, ERK = extracellular signal-regulated kinase, HIF-1α = hypoxic-inducible factor 1 alpha, JNK = c-Jun N-terminal kinase, MAPK = mitogen-activated protein kinase, NF-κB = nuclear factor kappa B, TGF = transforming growth factor, and VEGF = vascular endothelial growth factor.

**Table 9:** Melatonin’s effects as an adjunctive therapy in common therapeutics.

Treatment	Melatonin Concentration	Cell Type(s)	Effects in OS	References
Doxorubicin	10 mg of the MLT-nanocarrier	Saos-2 MG-63 human bone marrow mesenchymal stem cell, (hBM-MSC)	Improved DOX efficacy in cancer treatment and reduced toxicity.	[161]
Melatonin/HPβCD inclusion complex-loaded chitosan scaffolds	9 mM	MG-63	Time-dependant ↑ Apoptosis	[138]
Methotrexate	0.5 mmol/L 1 mmol/L 2 mmol/L 4 mmol/L 5 mmol/L	SaOS-2	↓ Cell activity ↑ Cells at G1 Cycle ↑ Apoptosis	[162]
Cisplatin-Methotrexate				
Cisplatin	19.74 µg/mL 179.1 µg/mL 7.72 µg/mL	MG-63	↑ Sensitivity ↓ BCL2 ↓ miR-181b ↑ CYLD ↑ CBX-7 ↑ p53 ↑ Apoptosis	[163]

Supporting benefits of melatonin in contemporary osteosarcoma therapeutics presented with cell types utilized in the studies and their effects in OS.

## Abbreviations

AANAT	Aralkylamine N-acetyltransferase
ABC	ATP-binding cassette transporter
ACTH	Adrenocorticotropic hormone
AD	Autosomal dominant
Akt	Protein kinase B
APC	Adenomatous polyposis coli
AR	Autosomal recessive
ATP	Adenosine triphosphate
Bcl2	B-cell lymphoma 2
Bcl-xL	B-cell lymphoma-extra large
BLM	Bloom syndrome protein
BMI	Body mass index
CDK2/4	Cyclin-dependant kinase 2/4
CDKN2A	Cyclin-dependant kinase inhibitor 2A
cGMP	Guanosine 3′,5′-cyclic monophosphate
CIC	Capicua transcriptional repressor
circRNA	Circular RNA
CK1	Casein kinase 1
c-Myc	Cellular myc
Co-F	Co-factor
COX	Cyclooxygenase
CREB	cAMP-response element binding protein
CSC	Cancer stem cell
CTGF	Connective tissue growth factor
DNMT	DNA methyltransferase
Dvl	Dishevelled protein
ECV	Extracellular vesicles
EGF	Epidermal growth factor
EMT	Epithelial–mesenchymal transition
ERK	Extracellular signal-regulated kinase
FGF	Fibroblast growth factor
GnRH	Gonadotropin hormone-releasing hormone
GSK-3β	Glycogen synthase kinase 3β
HIF-1α	Hypoxia-inducible factor 1 subunit alpha
HPβCD	2-Hydroxypropyl-beta-cyclodextrin.
IGF	Insulin growth factor
IL-2/6/12/34	Interleukin
INFγ	Interferon gamma
INK4A	CDK inhibitors superfamily
INOS	Inducible nitric oxide synthase
IP3	Inositol trisphosphate
JNK	Jun N-terminal kinase
Kif7	Kinesin family member 7
LA	Linoleic acid
LH	Luteinizing hormone
lncRNA	Long non-coding RNA
MAPK	Mitogen-activated protein kinase
M-CSF	Macrophage colony-stimulating factor
MCT1/4	Monocarboxylate transporter 1/4
MDM2	Murine double minute 2
MDR	Multidrug resistance
MeCP2	Methyl CpG binding protein 2
miR	MicroRNA
MMP-9	Matrix metallopeptidase-9
MSC	Mesenchymal stem cell
MT1	Melatonin receptor 1
MT2	Melatonin receptor 2
mTOR	Mechanistic (formerly “mammalian”) target of rapamycin
NCID	NOTCH intracellular domain
NF-κB	Nuclear factor kappa B
NOS	Nitric oxide synthases
OPG	Osteoprotegerin
OS	Osteosarcoma
OS-CSC	Osteosarcoma–cancer stem cell
OXPHOS	Oxidative phosphorylation
PDK	Pyruvate dehydrogenase kinase
PEDF	Pigment epithelium-derived factor
PGE2	Prostaglandin E2
P-gp	P-glycoprotein
PI3K	Phosphoinositide 3 kinase
PKC	Protein kinase C
PKG	Protein kinase G
PTEN	Phosphotase and tensin homolog
PTH	Parathyroid hormone
PTHR	Parathyroid hormone receptor
PTH-rp	Parathyroid-related peptide
RANKL	Receptor activator of nuclear factor kappa beta
Rb1	Retinoblastoma 1
RCT	Randomized clinical trial
RecQ	Recombination Q helicases
Rh0/ROCK	Rho-associated protein kinase
RNA	Ribonucleic acid
ROR	Retinoic acid receptor-related orphan receptor
ROS	Reactive oxygen species
R-SMAD	Receptor-regulated Smad
RUNX2	Runt-related transcription factor 2
SIRT3	Sirtuin 3
SMAD	Suppressor of mothers against decapentaplegic
SOD2	Superoxide dismutase 2
SOX5	S RY-Box transcription factor 5
STAT	Signal transducers and activators of transcription
SUFU	Suppressor of fused homolog
TCA	Tricarboxylic acid cycle
TCF/LEF	T-cell factor/lymphoid enhancer factor
TET	Ten-eleven translocation
TF	Transcription factor
TGF	Transforming growth factor
TGFβ	Transforming growth factor beta
TNFα	Tumor necrosis factor alpha
TP53	Tumor protein p53
TSC 1/2	Tuberous sclerosis 1/2
VEGF	Vascular endothelial growth factor
Wnt	Wingless/Integrated
WWOX	WW domain-containing oxidoreductase
